# Detection of *Trypanosoma cruzi*-infected triatomines in Iquitos: possible incipient colonisation in the largest metropolis of the Peruvian Amazon

**DOI:** 10.1590/0074-02760240257

**Published:** 2026-02-27

**Authors:** Fabiola Díaz-Soria, Karine Zevallos, Bryan Cabrera-Campos, Carmen Sinti-Hesse, Sebastià Jaume-Ramis, Wieslawa Alava-Flores, Darcy Acho-Bernuy, Silvia Vega-Chirinos, Jesús Pinto-Caballero, César Ramal-Asayag, Moisés Sihuincha, Claudia Paredes-Esquivel

**Affiliations:** 1 National Institute of Health, Centre for Tropical Diseases Research Hugo Pesce - Maxime Kuczynski, Iquitos, Peru; 2 Universidad Nacional de la Amazonia Peruana, Faculty of Human Medicine, Iquitos, Peru; 3 National Institute of Health, National Centre for Public Health, Laboratory of Chagas, Lima, Peru; 4 University of the Balearic Islands, Mediterranean Parasitology and Ecoepidemiology Research Group, Palma, Spain; 5 National Institute of Health, Laboratory of Entomology, Lima, Peru; 6 Hospital Regional de Loreto, Iquitos, Peru; 7 Arzobispo Loayza National Hospital, Lima, Peru; 8 Biomedical Research Networking Center for Infectious Diseases, Madrid, Spain

**Keywords:** Chagas disease, American trypanosomiosis, Trypanosoma cruzi, Peru, Amazon, emerging, triatominae

## Abstract

**BACKGROUND:**

While Chagas disease (CD) has been controlled in many South American regions, the Amazon basin has emerged as a new focus of transmission. Metropolitan Iquitos (Loreto, Peru), has recently shown signs of potential disease emergence.

**OBJECTIVE:**

To assess the risk of CD transmission by evaluating triatomine presence and infection rates in households across Iquitos and nearby communities.

**METHODS:**

Entomological surveillance was conducted in domestic and peridomestic environments, following blood donor screenings (2011-2018) that confirmed local *Trypanosoma cruzi* cases. Triatomines were collected manually, with traps, and through community reporting. Specimens were identified, epidemiological indices calculated, and infestation risk factors analysed using penalised logistic regression, receiver operating characteristic (ROC)/area under the curve (AUC) metrics, and exploratory principal component analysis (PCA).

**FINDINGS:**

Of 142 houses visited, 113 were inspected, yielding a density index of 0.26. Nine houses were infested, mostly in Loboyacu, with 29 adult triatomines collected — by *Rhodnius robustus* (89.7%) and *Panstrongylus geniculatus*. *T. cruzi* infection was confirmed, and palm roofs emerged as the strongest predictor of infestation [odds ratio (OR) > 16, p < 0.001].

**MAIN CONCLUSIONS:**

This first evidence of *T. cruzi* circulation in sylvatic triatomines within Metropolitan Iquitos highlights an emerging risk of CD. Although vectors remain scarce, palm-roofed houses, deforestation, and urban expansion may facilitate future transmission.

Chagas disease (CD) is a neglected tropical disease, endemic to 21 Latin American countries. Also known as American trypanosomiasis, it affects approximately 6-7 million people worldwide, causing around 12,000 deaths each year. Humans acquire the disease through contact with the faeces and urine of haematophagous triatomine insects carrying infective forms of *Trypanosoma cruzi*.^1^ ^,^ ^2^ In Peru, CD has historically been prevalent in the Southern region, with less information and resources devoted to understanding the transmission in the epidemiology of the Northern region of the country.^3^ ^,^ ^4^

The Amazon region has long been considered free from CD; however, enzootic transmission cycles have always known to occur in wild animals.^5^ Since the first report of an autochthonous case in French Guiana in 1941, CD has become established in nearly all Amazon countries.^6^ ^,^ ^7^ This shift has been associated to the globalisation of the Amazon region,^5^ as well as to human migration, extensive landscape transformation and deforestation.^8^ As a result, the epidemiology of CD in South America is changing dramatically, partly due to the intrusiveness of sylvatic triatomine insects into human dwellings, emerging as key vectors in new regions.^9^ This evolving scenario is particularly noticeable in Brazil, where 95% of CD cases reported in the country now occur in the Amazon, with oral transmission being the predominant form of infection.^10^

In Peru, the Amazon region is sparsely populated and is divided into two main areas: a mountainous highland jungle and a flat lowland jungle. Located in the northeastern part of this region, Loreto is the largest department and consists of eight provinces. The most populous of these is Maynas, which is home to Iquitos, the regional capital and largest city. While much of the vast primary forest in the Peruvian Amazon remains largely intact, it has steadily diminished since the early 2000s due to deforestation and habitat transformation. Between 2001 and 2024, Maynas province lost approximately 173.000 hectares of tree cover, representing a 1,3% decrease since 2000. Of this loss, 89,700 hectares were humid primary forest, which accounts for 54% of total deforestation and 0,71% decline in primary forest area. The main drivers of deforestation include shifting agriculture, permanent agriculture, and selective logging. Despite these pressures, Maynas retains extensive forest cover.^10^

Although the Peruvian Amazon region is considered a hotspot for various infectious diseases, particularly vector-borne illnesses,^11^ CD has been reported only sporadically in the region.^7^ ^,^ ^12^ Since 2006, however, CD has been continuously reported in the Loreto population, with the first case identified in an indigenous child from the westernmost province, Datem del Marañón. Between 2007 and 2009, five additional cases were documented in the same province, all involving indigenous children.^13^ In 2008, an acute *T. cruzi* infection was also reported in a rural town within the Pebas, district of Mariscal Ramón Castilla province, located near the Brazil border, about 160 km from Iquitos.^14^ In 2014, a paediatric case was confirmed in the Mazán district, just 33 km from Iquitos.^7^ More recently, two human cases were reported in Metropolitan Iquitos: one in 2015 involving an 8-year-old child from the San Juan Bautista district^7^ and another in 2019, identified during serological surveillance of pregnant women^4^ (Fig. 1).

**Fig. 1:**
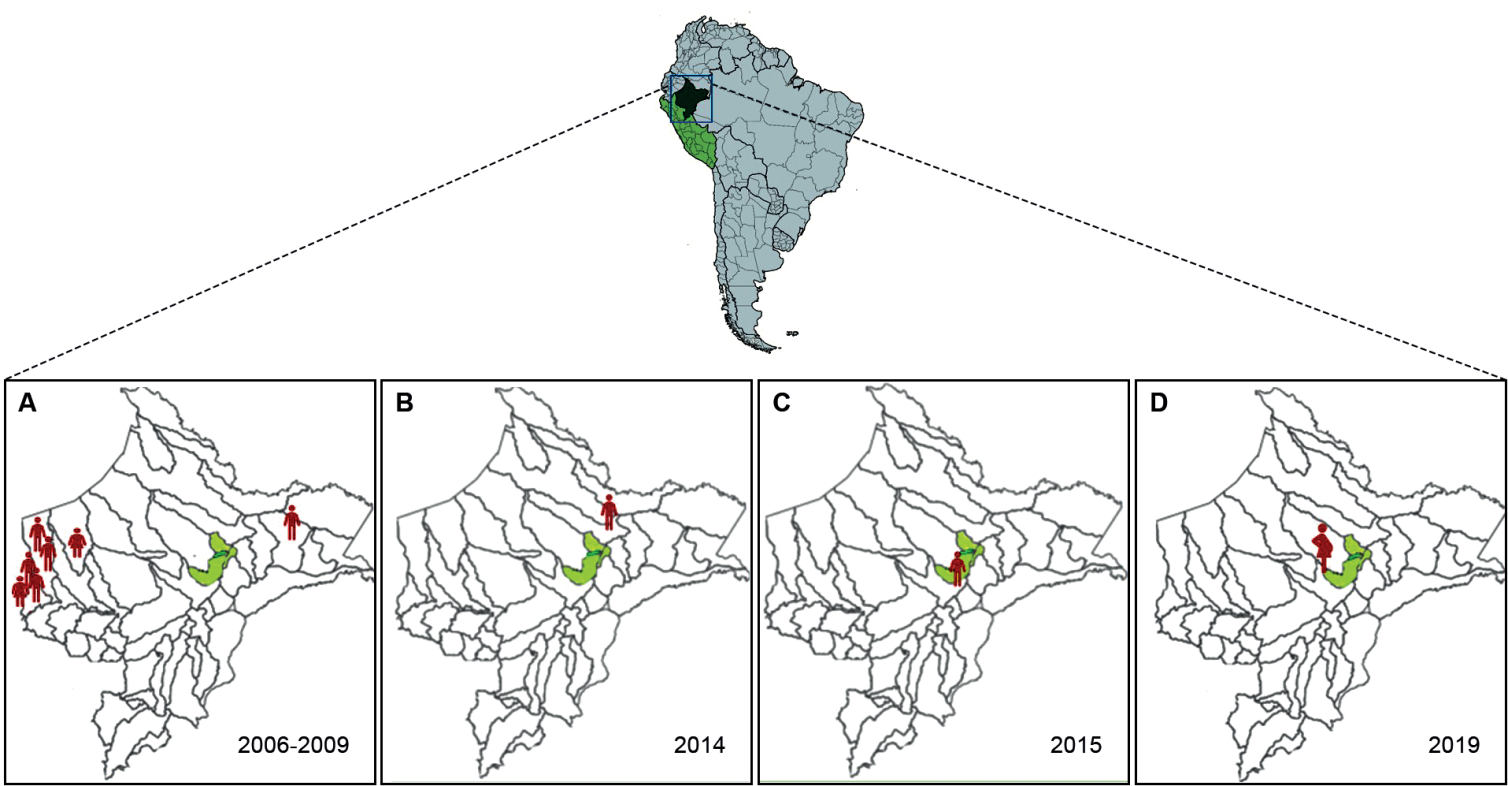
geographic distribution and temporal evolution of Chagas disease (CD) cases in the Loreto department (dark green area), in Peru (green area), prior to this study. (A) Between 2006 and 2009, six confirmed acute cases occurred in indigenous children across various districts in Datem del Marañón Province (12), along with one pediatric case in Pebas, Mariscal Ramón Castilla Province (13); (B-C) during 2014-2015, two pediatric cases were reported: one in Mazán district (B) and one in San Juan Bautista district, located on the outskirts of Metropolitan Iquitos (C) (7); (D) in 2019, a seropositive case was detected in a pregnant woman in 2019 (4). Metropolitan Iquitos, with a population exceeding 460,000, is highlighted in light green within the Loreto department.

The growing risk of CD transmission in the Peruvian Amazon has been previously documented;^13^ however, data on the role of triatomine vectors remain limited.^15^ To facilitate the early identification of active transmission in Iquitos and surrounding areas, we conducted a comprehensive entomological surveillance of triatomine species that invade human settlements. Target areas were identified after a thorough review of the epidemiological information of positive cases in blood donors, as reported by the Peruvian Ministry of Health in Loreto.

## MATERIALS AND METHODS

*Study area and population* - The Peruvian Amazon region occupies 60% of the country's territory but is home to only 5% of its population. This study was conducted in the province of Maynas, in northeastern Peru (Fig. 1). Iquitos, its capital city (73.2º W, 3.7º S, 120 m), is the most densely populated metropolis in the Peruvian Amazon rainforest and the sixth most populated city in the country. Located near the confluence of the Marañón and Ucayali rivers, Iquitos is geographically isolated, accessible only by air or river, and home to approximately 460,000 people. Metropolitan Iquitos comprises four districts: Iquitos, Punchana, San Juan Bautista, and Belén. The metropolis is highly diverse, with rural, periurban, and urban settlements.

*Baseline* - Between 2011 and 2018, screening tests for anti-*T. cruzi* antibodies were conducted using Chagatest ELISA® (Wiener Lab, Rosario, Argentina) as part of the blood donor surveillance program at the Hospital Regional de Loreto in Iquitos. During this period, 12,605 blood samples were analysed, of which 46 tested reactive for *T. cruzi*. Due to the occasional limitations in Chagatest reliability, positive samples were sent to the National Institute of Health (Instituto Nacional de Salud - INS) Chagas reference laboratory in Lima for seropositivity confirmation through indirect immunofluorescence and Inmunoblot IgG according to the institute guidelines.^16^ CD seropositivity was confirmed in eight patients, six of whom were residents of metropolitan Iquitos, while two were from Mazán and Fernando Lores districts, within the Loreto department [Supplementary data (Table I)].

*Sampling approach* - Before sampling, this study was reviewed and approved by Peruvian health authorities from the National Institute of Health in Lima, Perú. Sampling sites included human settlements in Metropolitan Iquitos, constituted by districts: Iquitos, San Juan Bautista, Belen and Punchana. A convenience sampling strategy was implemented in two stages. First, triatomine collection focused on the residences of the eight patients with confirmed CD. In the second stage, collections were extended to neighbouring houses — defined as those adjacent to the confirmed cases. Additionally, flyers featuring images of triatomines and the principal investigator's phone number were distributed throughout the districts. A structured questionnaire was not used; instead, open-ended questions were asked to determine whether residents had observed triatomines in recent months. Collections were also carried out in other homes within the same districts where residents reported sightings of triatomines. The entomological surveillance was extended to Puerto Abeja (Mazán district) where a case had previously been detected.^7^

Insect surveillance was conducted following verbal consent from the heads of households. When home-owners were unavailable, adjacent houses were sampled based on information provided by neighbours, with a standardised capture effort of one person-hour per dwelling. In domestic (intradomicile) environments, triatomines were manually collected following the methodology described by Paredes-Esquivel et al.^3^ For peridomestic (outdoor) environments, light traps consisting of white blankets (2 × 2 m) illuminated by white incandescent bulbs were used to attract insects. To enhance sampling success, live bait traps were also employed. These traps consisted of open cages (30 cm x 15 cm x 15 cm) containing an animal as bait — either BALB/c mice or small chickens in the case of Mazán. The cages were lined with double-sided adhesive tape, ensuring that insects adhered to the tape when they entered to feed. Both traps were left overnight, adhering to the guidelines outlined by the Ministry of Health of Peru.^17^ Additionally, homeowners were provided with small plastic cages and tweezers for the manual collection of specimens. The cages were constructed from plastic flasks with a small opening covered by tulle fabric to allow airflow. Live specimens were placed inside the cages, and research staff visited the households the following day to retrieve the collected specimens. Collected triatomines were identified in the laboratory, using the identification keys provided in the Manual de procedimientos de identificación de triatominos del Perú.^17^ Specimen collection was conducted intermittently between 2018-2019, as conditions allowed, rather than through continuous surveillance.

*Entomo-epidemiological indices and data analysis* - The following epidemiological indices of CD transmission were calculated, according to the established guidelines: density index (number of captured triatomines/number of examined houses); infestation index (number of infested houses/ number of examined houses x 100), disaggregated in intra and peridomicile, house infection index (number of houses with infected triatomines/number of explored houses) and natural infection index (number of *T. cruzi* positive triatomines/number of examined triatomines x 100).

Data analysis to identify factors associated with the intradomicile presence of triatomine vectors in the rural area of Iquitos (Peru) was conducted using R.^18^ A variable was considered statistically significant when p < 0.05. Confirmed triatomine infestation in the houses (infested_houses) was used as a binary response variable (0 = not infested, 1 = infested). Prior to model fitting, potential collinearity among predictors was evaluated by calculating the variance inflation factor (VIF) using the car package.^19^ Among the variables recorded during the survey (Table I), those describing similar ecological features (such as different types of vegetation or animal presence) were aggregated into combined indices (vegetation_total and animals_nearby_total) to reduce redundancy. Similarly, wall categories with sparse representation were grouped into a new "other" category. Given the small number of positive cases (see Results) and the risk of overfitting, only variables with clear ecological relevance were retained in the final models.

**TABLE I:** Original variables recorded during household visits and their corresponding grouped variables, including variable type, levels, and a brief description

Variables	Type	Levels	Description
Infested_houses (response variable)	Binomial	0, 1	Presence or absence of triatomines identified by researchers
Roof_material	Categorical	Calamine, palm	Material used for roof construction
Wall_material	Categorical	Wood, brick, costal, without walls	Material used for wall construction
Wall_material_grouped	Categorical	Wood, brick, other	Same as above but grouping the levels costal and without walls into "other" to reduce complexity
Floor_material	Categorical	Wood, earth, concrete	Material used for floor construction
Mattress_material	Categorical	Foam, no mattress, spring, thatch	Material used as mattress
Cracks_observed	Binomial	0, 1	If cracks were observed inside the house
Vegetation_present	Binomial	0, 1	If at least palms, other trees or scrublands were present in the area
Vegetation_total	Categorical	0, no vegetation; 1, palm, other trees or scrublands; 2, two types of mentioned vegetation; 3, the three types of vegetation are present in the area	Same as above but taking into consideration the type of vegetation
Have_animals	Binomial	0, 1	If owners have pet animals or animals for food such as chickens, etc.
Animal nests	Binomial	0, 1	If animal nests were observed by owners around the house
Outside_animals_present	Binomial	0, 1	If owners did observe any kind of animal around the house
Animals_nearby_total	Categorical	0, no animals observed 1, animal or nests observed 2, animals and nests observed in the area	Same as the two mentioned before but considering each case.

In preliminary analysis, binomial logistic regression models (GLM with link = logit) were tested but exhibited separation problems, unstable estimates, and excessively large standard errors, mainly due to the very low number of infested houses. To avoid this, we employed penalised logistic regression with Firth's correction, using the R package logistf.^20^ This method provides unbiased and more reliable parameter estimates in the presence of low prevalence and data separation. Two main models were fitted: one structural model, including only housing construction variables (roof and wall material), and a combined model, including both structural and environmental predictors (vegetation and animal presence). Model discrimination was assessed using receiver operating characteristic (ROC) curves and the area under the curve (AUC) parameter, computed with the pROC package.^21^ In addition to regression modelling, we conducted a principal component analysis (PCA) to explore overall patterns of association among household characteristics. All structural and environmental variables were included in the PCA after standardisation. Biplots of the first two principal components were generated using the ggplot2 package.^22^ The PCA was intended as an exploratory tool to visualise multivariate relationships, rather than for variable selection.

*Detection of T. cruzi infection in triatomines* - Faecal drops were obtained from alive triatomines by abdominal compression as described by Paredes-Esquivel et al.^3^ When this technique failed, intestinal content was obtained by abdomen puncture. Characteristic mobile flagellate forms were investigated using an optical microscope (100x and 400x magnifications) and morphologically identified as trypanosomes.

Subsequently, all collected insects were subjected to molecular analyses in the CD reference laboratory at the National Institute of Health in Lima. Individual triatomine's midgut contents were obtained through necropsy. Total DNA was extracted using PureLink® Genomic DNA Mini Kit (Invitrogen™), following manufacturer instructions. *T. cruzi* infection was detected by the simultaneous amplification of two molecular markers: the highly repetitive genomic satellite DNA, using primers TCZ1 (5'-CGAGCTCTTGCCCACACGGGTGCT-3') and TCZ2 (5'-CCTCCAAGCAGCGGATAGTTCAGG-3') and the minicircle variable region of the kinetoplastid DNA, using primers S35 (5'-AAA TAA TGT ACG GGK GAG ATG CAT GA-3') and S36 (5'-GGGTTCGATTGGGGTTGGTGT-3').^23^ ^,^ ^24^ *T. cruzi* lineages were characterised by the amplification of the mini-exon, 18S, 24S genes as described by Brisse et al.^25^ Polymerase chain reaction (PCR) products were separated in a 2% agarose gel electrophoresis and visualised with a ultraviolet (UV) transilluminator.

*Ethics* - The experiments were conducted in strict adherence to the guidelines established by the Peruvian National Institute of Health, ensuring compliance with all ethical and procedural standards for research.

## RESULTS

**Fig. 2:**
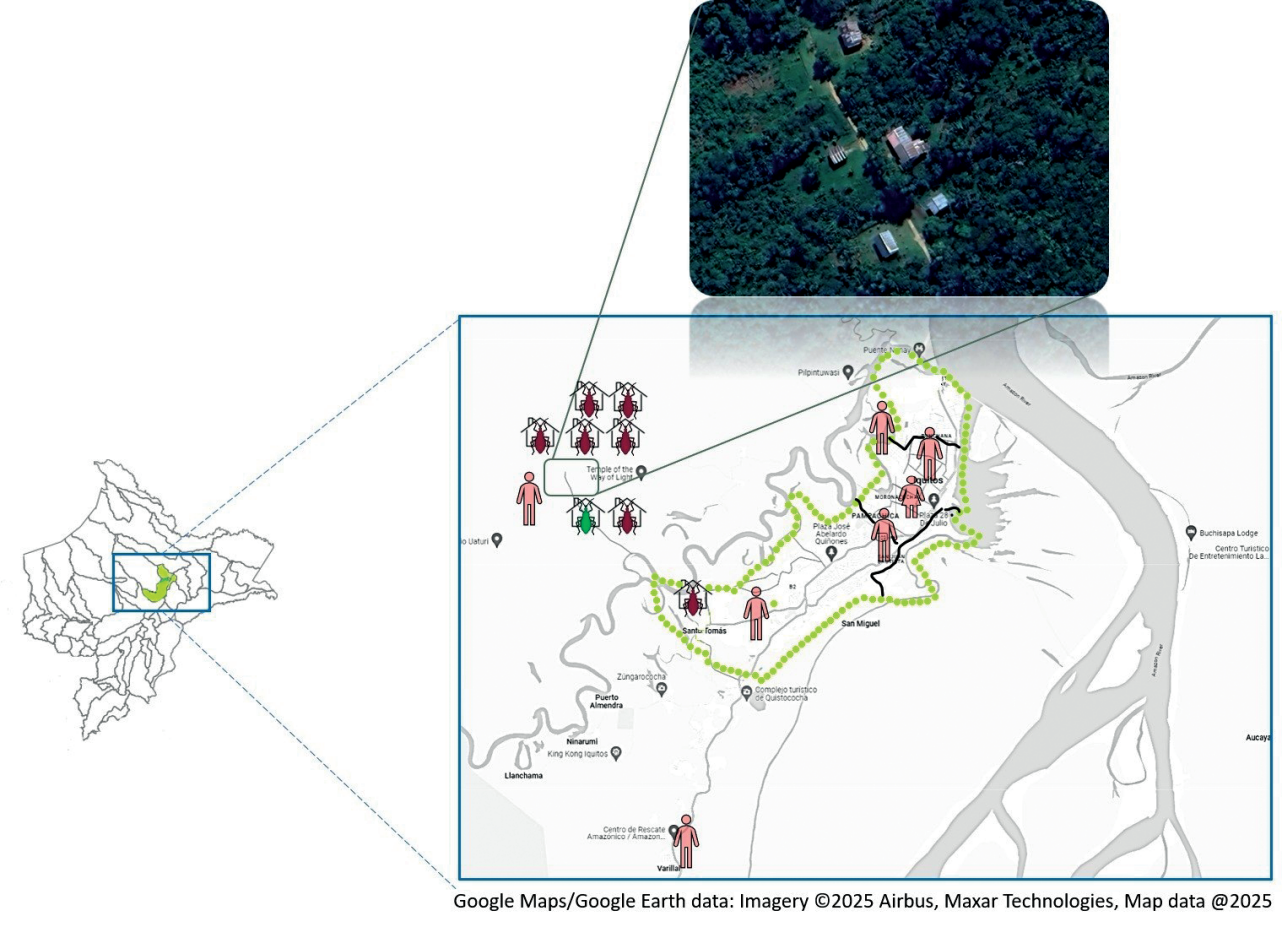
epidemiological situation of Chagas disease (CD) in Iquitos. Houses where *Trypanosoma cruzi*-infected triatomines are represented in purple, while those with non-infected triatomines are shown in green. Confirmed seropositive cases in the city, detected by the Ministry of Health of Peru, are also depicted. Below: an aerial photograph of Loboyacu village, in the Iquitos district, where most of the positive houses were located. Please notice that although Loboyacu village appears to be outside the boundaries of Iquitos, this is considered a newly incorporated community. District boundaries are outlined in black: Punchana to the north, Iquitos in the centre, San Juan Bautista to the south, and Belén to the east.

*Insects* - Of the 142 houses visited, 113 were accessible and thoroughly inspected, yielding a triatomine density index of 0.26. The inspected households were distributed across several districts: 62 in San Juan Bautista, 29 in Iquitos, 19 in Punchana, and two in Belén. Additionally, one house in Puerto Abeja (Mazán District) was included in the surveillance based on a previously reported positive case in the area. The majority of infested houses (n = 7) were located in Loboyacu (Iquitos District), a rural village on the outskirts of Iquitos (Fig. 2). Of the eight confirmed patients' residences, only six were available for survey, as two patients had either relocated or could not be contacted.

A total of 29 triatomine adult specimens were collected in the nine houses between 2018-2019. During the first phase of the study, 67 households were visited and inspected, of which one household in Puerto Abeja (Mazán District) was found to be infested, yielding two triatomine specimens. In the second phase, 46 households were inspected, with triatomine infestations detected in eight households. A total of 27 triatomines were collected during this phase — 26 specimens from Loboyacu and one specimen from Santo Tomás (San Juan Bautista District) (Table II). While one specimen was provided directly by a homeowner, the remaining specimens were collected by the research team. Within Loboyacu, triatomines were found in the intradomicile areas of five houses, the peridomicile area of one house, and in both intra- and peridomicile areas of one additional house.

*Rhodnius robustus* (89.7%) was the most common vector species found in domestic and peridomiciliary areas across the three sampling locations. Three *Panstrongylus* specimens (10.3%) were collected from a chicken corral in a single house in Loboyacu village (Table II). In general, 16 of the 29 triatomines were collected from inside the domicile and 13 from the peridomicile. The domestic (intradomicile) infestation index was 6.19, while the peridomiciliary index was 2.65. Penalised logistic regression (Firth's correction) models showed similar results (Table III). In the structural model (which included roof and wall material), the presence of palm roofs was strongly and significantly associated with infestation. Households with palm roofs were over 20 times more likely to be infested compared with those with calamine roofs [odds ratio (OR) = 20.5, confidence interval (CI) 95%: 3.8-211.4, p < 0.001]. Wall material did not show a significant effect, with wide CI reflecting the small number of infested houses.

In the combined model (which additionally included vegetation and animal presence variables), palm roofs remained significantly associated with infestation (OR = 16.2, CI95%: 3.1-163.4, p < 0.001). Neither vegetation nor animal presence indices showed significant associations with infestation, and their CIs were extremely wide. Both structural and combined models showed high and similar discrimination ability (AUC = 0.90 and 0.92, respectively). Overall, the results consistently identified palm roofs as the main factor associated with triatomine infestation in the studied households, while environmental variables did not contribute significantly in this case.

**TABLE II:** Distribution of triatomine species collected in Mazán and Iquitos districts, Loreto

S. code	Species	Locality	District	Infest. house	Intradomicile	Peridomicile	Sex	Morf	PCR	*T. cruzi* lineage
T1	*Rhodnius robustus*	Puerto Abeja	Mazán	House 1	bed		Male	+	+	TcI
T2	*Rhodnius robustus*	Puerto Abeja	Mazán	House 1	bed wall		Female	+	+	undetermined
T3	*Rhodnius robustus*	Loboyacu	Iquitos	House 2	living room		Male	-	+	undetermined
T4	*Rhodnius robustus*	Loboyacu	Iquitos	House 2	living room		Male	-	+	undetermined
T5	*Rhodnius robustus*	Loboyacu	Iquitos	House 2	bed		Female	-	+	undetermined
T6	*Rhodnius robustus*	Loboyacu	Iquitos	House 3		chicken corral	Female	-	-	-
T7	*Rhodnius robustus*	Loboyacu	Iquitos	House 3		chicken corral	Female	-	+	undetermined
T8	*Panstrongylus geniculatus*	Loboyacu	Iquitos	House 3		chicken corral	Female	+	+	undetermined
T9	*Rhodnius robustus*	Loboyacu	Iquitos	House 3		chicken corral	Male	-	-	-
T10	*Panstrongylus geniculatus*	Loboyacu	Iquitos	House 3		chicken corral	Male	+	+	TcI
T11	*Panstrongylus geniculatus*	Loboyacu	Iquitos	House 3		chicken corral	Female	+	+	undetermined
T12	*Rhodnius robustus*	Loboyacu	Iquitos	House 3		chicken corral	Female	+	+	TcI
T13	*Rhodnius robustus*	Loboyacu	Iquitos	House 3		chicken corral	Male	-	+	undetermined
T14	*Rhodnius robustus*	Loboyacu	Iquitos	House 3		chicken corral	Female	-	+	undetermined
T15	*Rhodnius robustus*	Loboyacu	Iquitos	House 3		chicken corral	Female	-	+	TcI
T16	*Rhodnius robustus*	Loboyacu	Iquitos	House 3		chicken corral	Female	+	+	TcI
T17	*Rhodnius robustus*	Loboyacu	Iquitos	House 4	mosquito net		Female	+	+	undetermined
T18	*Rhodnius robustus*	Loboyacu	Iquitos	House 5	bed		Female	-	+	undetermined
T19	*Rhodnius robustus*	Loboyacu	Iquitos	House 5	bedroom wall		Female	-	-	-
T20	*Rhodnius robustus*	Loboyacu	Iquitos	House 5	palm ceiling		Male	+	+	undetermined
T21	*Rhodnius robustus*	Loboyacu	Iquitos	House 5	living room		Male	+	+	undetermined
T22	*Rhodnius robustus*	Loboyacu	Iquitos	House 6	living room		Male	-	-	-
T23	*Rhodnius robustus*	Loboyacu	Iquitos	House 6	living room		Male	-	-	-
T24	*Rhodnius robustus*	Loboyacu	Iquitos	House 6	bedroom wall		Female	-	-	-
T25	*Rhodnius robustus*	Loboyacu	Iquitos	House 7	bed		Male	+	+	TcI
T26	*Rhodnius robustus*	Loboyacu	Iquitos	House 8	living room		Male	-	+	undetermined
T27	*Rhodnius robustus*	Loboyacu	Iquitos	House 8	living room		Male	-	+	undetermined
T28	*Rhodnius robustus*	Loboyacu	Iquitos	House 8		chiken corral	Male	-	+	TcI
T29	*Rhodnius robustus*	Santo Tomas	S. Juan Bautista	House 9		tree	Male	+	+	TcI

**TABLE III:** Summary results of the penalized logistic regression (Firth's correction) models. The structural model included only housing construction variables, while the combined model included both structural and environmental predictors

Structural model (AUC = 0.90)			
Variables	OR	CI95%	p-value
Roof material = palm	20.5	3.8 - 211.4	< 0.001
Wall material = other	1.28	0.005 - 362.3	0.91
Wall material = wood	2.11	0.11 - 311.7	0.63

Combined model (AUC = 0.92)			
Variables	OR	CI95%	p-value
Roof material = palm	16.2	3.1 - 163.4	< 0.001
Wall material = other	1.87	0.006 - 584.2	0.80
Wall material = wood	1.55	0.08 - 224.7	0.78
Total vegetation	0.89	0.25 - 406.1	0.91
Animals nearby total	2.82	0.47 - 1124.6	0.32

AUC: area under the curve; CI: confidence interval; OR: odds ratio.

**Fig. 3:**
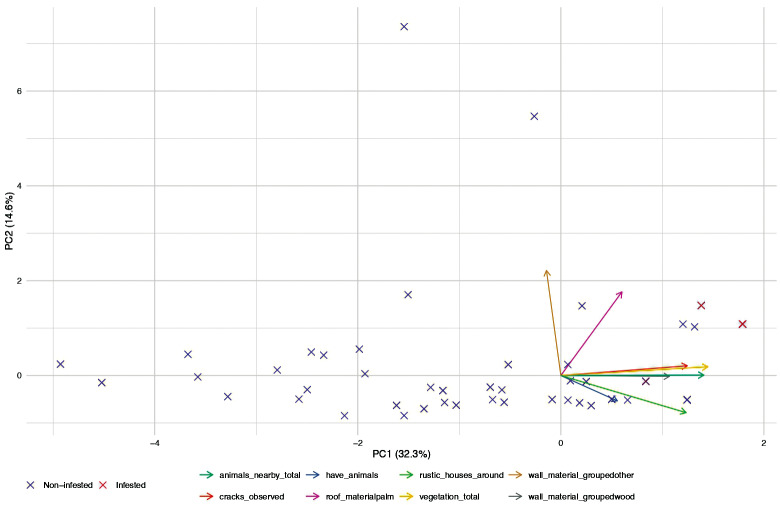
principal component analysis (PCA) to explore overall patterns of association among household characteristics.

The PCA showed that the first two components explained 46.9% (Fig. 3) of the total variance in household characteristics. Infested and non-infested houses did not cluster separately, being scattered throughout the ordination space and largely overlapped with non-infested households. Structural and environmental variables contributed to overall variation among households, but no specific combination of variables clearly separated infested from non-infested.

In the Iquitos district, infected triatomines were present in six of the seven infested houses, located in the rural village of Loboyacu (Fig. 2). In San Juan Bautista district, a single triatomine was collected from a palm tree in the peridomicile area of a house in the periurban locality of Santo Tomás. This specimen tested positive for *T. cruzi* marking the only positive detection among the 62 houses surveyed in San Juan Bautista (Fig. 2).

Overall, house infection index in Iquitos city was 6,19% and natural infection index among collected triatomines was 24,14%. The isolated house from Mazán resulted positive for *T. cruzi* but this house was excluded from the entomological indices analysis due to its location outside Iquitos.

## DISCUSSION

In parallel to the success in controlling American Trypanosomiasis in many regions, the Amazon basin has raised as a new epidemiological focus of the disease in the last two decades. Human migration, mammal's displacement, deforestation and habitat transformation has been associated with its emergence.^6^ ,8 This study presents the results of comprehensive entomological surveillance conducted in domestic and peridomestic settings of houses in various localities within Metropolitan Iquitos (Loreto), the largest metropolis in the Peruvian Amazon.

We report the detection of *T. cruzi* in 79.31% of the sylvatic triatomines — *R. robustus* and *P. geniculatus* — collected from domestic and peridomestic settings across Metropolitan Iquitos (Loreto). This marks the first confirmed report of *T. cruzi* circulation in these, or in any other triatomine species within Metropolitan Iquitos and its surrounding communities. Both *R. robustus* and *P. geniculatus* species are widely distributed in northern Peru^26^ and have previously been associated with human cases in Loreto. Specifically, *P. geniculatus* was found in the dwelling of a reported case in the Pebas district,^15^ and the strictly sylvatic species *R. robustus* has been identified in the Datem del Marañón province.^13^ However, in neither case, *T. cruzi* infection could be confirmed. Comparable patterns of high *T. cruzi* infection rates in these triatomine species have been documented in the Bolivian Amazon,^27^ suggesting that such sylvatic vectors may play a significant role in the transmission dynamics of CD s throughout the Amazon region.^8^

One important limitation of this study is the lack of molecular analysis for the identification of triatomine specimens. This is particularly relevant in the case of *R. robustus*, which is morphologically very similar to other species within the genus — most notably *Rhodnius prolixus* and *Rhodnius montenegrensis*.^28^ Although *R. prolixus* has not previously been recorded in Peru,^17^ *R. montenegrensis* has been recently identified in a locality near the border with Bolivia and Brazil,^29^ highlighting he importance of molecular tools to accurately distinguish among these closely related species. Mitochondrial markers, such as cytochrome b, have proven effective in distinguishing between these closely related species and should be incorporated into future studies.^30^ Additionally, a limitation related to parasite genotyping should be noted. Only 35% of the *T. cruzi*-positive samples were successfully classified as TcI, likely due to low parasite loads in some insects and variability in the quality of extracted DNA, which prevented clear amplification in all lineage-typing assays.

Our surveillance efforts indicate that triatomines remain relatively scarce in Iquitos, with only nine out of 113 examined houses found to be infested. However, we detected an infected triatomine in a palm tree at a residence in the Santo Tomás neighbourhood, demonstrating that *T. cruzi* circulates in insect vectors not only in isolated villages like Loboyacu but also in more densely populated urban areas (Fig. 2). Moreover, the recent reports of positive human cases in Iquitos and the seroprevalence observed in blood donor screenings^4^ ,7 may signal an emerging risk of increased CD transmission. Nonetheless, in the absence of prior systematic studies, these findings should be interpreted cautiously. Although only seven houses were found to be infested during our study, 31 homeowners reported sightings of kissing bugs in their homes [Supplementary data (Table II)], suggesting that triatomine presence may be underdetected by the current sampling approach. Increasing the frequency of visits per site and extending the sampling period through systematic surveillance could improve detection rates and provide a more accurate picture of triatomine distribution and infestation levels. Continued and enhanced surveillance is therefore essential to better assess the evolving epidemiological situation in Iquitos.

Overall, roof type consistently emerged as the strongest predictor across models. However, the low number of infested houses limits the precision of statistical estimates, leading to wide CIs (Table III). Houses with palm roofs were over 20 times more likely to be infested compared to those with calamine roofs. Palms have been demonstrated to be excellent habitats for sylvatic triatomines (particularly *Rhodnius* spp.), as these insects can invade nearby houses by flying from the trees.^31^ Historically, indigenous populations in the Amazon area do not raise domestic animals;^5^ however, this trend appears to be changing due to migration.^6^ Interestingly, palm trees located at the border between human settlements and the forest are more likely to be infested by triatomines than those located deeper within the forest.^32^ Consequently, it is logical that most triatomines were collected in the rural village of Loboyacu (Fig. 2). Deforestation in metropolitan Iquitos and its surrounding areas has increased in recent years, partly due to urban growth.^33^ Given the strong relationship between triatomines intrusiveness and deforestation, particularly *P. geniculatus*,^34^ the increasing deforestation may also elevate the risk of human transmission in Iquitos.

Chagas disease transmission has unique characteristics in the Amazon, where oral transmission has shifted from being rarely reported to a significant route of infection. In the Amazon region of Brazil, oral transmission has been associated with several outbreaks of acute CD.^35^ For instance, in 2006, a single outbreak involving 178 cases of CD were linked to the consumption of açaí juice (*Euterpe oleracea*) in Brazil.^36^ In the Peruvian Amazon region, the ingestion of masato — a local fermented beverage made from cooked manioc and cane juice — was suspected to be associated with an outbreak.^13^ Food-borne CD outbreaks via oral transmission are known to result in higher morbidity and mortality.^1^ Therefore, authorities should implement effective preventive measures, similar to those adopted by the Brazilian Agricultural Research Corporation, which has established specific guidelines for pre-treating açaí fruits before consumption to prevent oral transmission of CD.^37^

Early detection of CD emergence is crucial for the timely implementation of effective prevention, control, and treatment strategies. Benznidazole and Nifurtimox, for instance, are highly effective if administered immediately after infection, achieving cure rates of up to 100%.^38^ In the Amazon region, where transmission dynamics are complex, robust and sustained surveillance is essential to identify areas of active transmission. A comprehensive, multi-prolonged approach — including routine screening of blood and organ donors, entomological monitoring, and targeted vector control measures^9^ ,39 — is essential to detect and manage emerging hotspots. Such efforts are particularly vital in the Peruvian Amazon basin, where limited coordinated interventions have been implemented. Strengthening surveillance and control in this region could significantly reduce the risk of human cases and help prevent further spread of the disease.

## SUPPLEMENTARY MATERIALS

Supplementary material

## Data Availability

The contents underlying the research text are included in the manuscript.
