# Supplementary material for: Detection of Trypanosoma cruzi-infected triatomines in Iquitos: possible incipient colonisation in the largest metropolis of the Peruvian Amazon
Source: Mem Inst Oswaldo Cruz. 2026 Feb 27;121:e240257. doi: 10.1590/0074-02760240257 (PMC12948030; doi:10.1590/0074-02760240257)
Supplement: Supplementary material [file 1678-8060-mioc-121-e240257-s1.pdf]

TABLE I

Epidemiological data from 113 of the 142 houses included in this study

Basic information (patient)					Address				Presence of triatomines						Chagas disease test		
House code	Age	Education level	Place of birth	Sex	Department	Province	District	Coordinates	Houses w/ triatomines intradom.	Triatomine intradom. Positive(N)	triatomines intradomocile negative(N)	Houses with triatomines peridomicile	triatomines peridomicile positive(N)	triatomines peridomicile negative(N)	Positive serology (blood donors)	Positive INS	Second test date (INS)
1	29,0	secondary school	Pevas	M	Loreto	Maynas	Iquitos	3°43’51’’ S, 73°16’19’’ W	0	0	0	0	0	0	Y	**Y**	05/04/2018
2	-	-	Iquitos	-	-	-	-	not found	-	-	-	-	-	-	Y	0	0
3	49,0	secondary school	Iquitos	M	Loreto	Maynas	San Juan	3°53’41’’ S, 73°20’53’’ W	0	0	0	0	0	0	Y	**Y**	09/06/2018
4	42,0	University	Lima - Norte	M	Loreto	Maynas	Punchana	3°43’36’’ S, 73°14’30’’ O	0	0	0	0	0	0	Y	N	26/04/2018
5	-	-	Indiana	-	-	-	-	not found	-	-	-	-	-	-	Y	0	0
6	41,0	University	Iquitos	M	Loreto	Maynas	Iquitos	3°44’45’’ S, 73°15’11’’ w	0	0	0	0	0	0	Y	**Y**	24/04/2018
7	58,0	secondary school	Jepelacio	M	Loreto	Maynas	San Juan	3° 56′33.67′′S, 73°22′1.60′′O	0	0	0	0	0	0	Y	N	22/05/2018
8	25,0	University	Iquitos	M	Loreto	Maynas	San Juan	3°50’49’’ S, 73°20’24’’ O	0	0	0	0	0	0	Y	N	15/05/2018
9	not found	-	Iquitos	-	-	-	-	-	-	-	-	-	-	-	Y	**Y**	14/10/2014
10	not found	-	Lagunas Rio Huallaga	-	-	-	-	-	-	-	-	-	-	-	Y	0	0
11	not found	-	Sta Rosa	-	-	-	-	-	-	-	-	-	-	-	Y	0	0
12	not found	-	Pucallpa	-	-	-	-	-	-	-	-	-	-	-	Y	0	0
13	not found	-	Iquitos	-	-	-	-	-	-	-	-	-	-	-	Y	0	0
14	31,0	technical	Iquitos	M	Loreto	Maynas	Iquitos	3°44’22.1”S 73°14’31.8”O	0	0	0	0	0	0	Y	N	05/04/2018
15	41,0	Primary school	Marañon, Pto. Auxilio	M	Loreto	Maynas	San Juan	3°47’43’’ S, 73°18’51’’ O	0	0	0	0	0	0	Y	N	08/05/2018
16	48,0	University	Lima	M	Loreto	Maynas	Iquitos	3°44’8’’ S, 73°15’42’’ O	0	0	0	0	0	0	Y	N	17/05/2018
17	not found	-	Lagunas	-	-	-	-	-	-	-	-	-	-	-	Y	0	0
17	49,0	University	Requena	M	Loreto	Maynas	Belén	3°46’21.8”S 73°15’46.2”O	0	0	0	0	0	0	Y	N	05/04/2018
18	not found	-	Iquitos	-	-	-	-	-	-	-	-	-	-	-	Y	0	0
20	31,0	secondary school	Juancito Rio Ucayali	M	Loreto	Maynas	Belén	3°46’08.8”S 73°15’32” O	0	0	0	0	0	0	Y	N	25/04/2018
21	not found	-	Iquitos	-	-	-	-	-	-	-	-	-	-	-	Y	0	0
22	31,0	secondary school	Iquitos	F	Loreto	Maynas	Iquitos	3°44.334′ S 073°15.000′ O	0	0	0	0	0	0	Y	N	24/04/2018
23	not found	-	Tamishiyacu	-	-	-	-	-	-	-	-	-	-	-	Y	0	0
24	not found	-	Moyobamba	-	-	-	-	-	-	-	-	-	-	-	Y	0	0
25	37,0	secondary school	Iquitos	F	Loreto	Maynas	San Juan	S 3°53′39” W73°20′54”	0	0	0	0	0	0	0	0	0
26	50,0	Primary school	Iquitos	F	Loreto	Maynas	San Juan	S 3°53′42” W 73°20′52”	0	0	0	0	0	0	0	0	0
27	62,0	secondary school	Alto Tapiche Santa Elena	M	Loreto	Maynas	San Juan	3°53′43′′ S 73°20′52′′ W	0	0	0	0	0	0	0	0	0
28	50,0	secondary school	Alto Tapiche Santa Elena	F	Loreto	Maynas	San Juan	3°53′44′′ S 73°20′53′′ W	0	0	0	0	0	0	0	0	0
29	41,0	secondary school	Iquitos	M	Loreto	Maynas	Iquitos	3°44’35.6”S 73°15’01.0”O	0	0	0	0	0	0	Y	N	08/05/2018
30	not found	-	Iquitos	-	-	-	-	3°71’439’’ S, 73°24’698’’ O	-	-	-	-	-	-	Y	0	0
31	35,0	Primary school	San Roman - Rio Napo	F	Loreto	Maynas	Punchana	03°43.545′, 073°15.544′	0	0	0	0	0	0	0	0	0
32	not found	-	Iquitos	-	-	-	-	-	-	-	-	-	-	-	Y	0	0
33	not found	-	Iquitos	-	-	-	-	-	-	-	-	-	-	-	Y	0	0
34	31,0	Primary school	Urarinas	F	Loreto	Maynas	San Juan	3°53′41′′ S 73°20′41′′ W	0	0	0	0	0	0	0	0	0
35	62,0	Primary school	Comunidad Bagazan	F	Loreto	Maynas	San Juan	3°53′50′′ S 73°20′47′′ W	0	0	0	0	0	0	0	0	0
36	44,0	secondary school	Iquitos	F	Loreto	Maynas	San Juan	3°48′37′′S 73°18′57′′W	0	0	0	0	0	0	0	0	0
37	32,0	secondary school	San Martin	F	Loreto	Maynas	San Juan	3°48′38′′S 73°18′58′′W	0	0	0	0	0	0	0	0	0
38	76,0	Primary school	Iquitos	M	Loreto	Maynas	San Juan	3°48′24′′S 73°19′32′′W	0	0	0	0	0	0	0	0	0
39	75,0	Primary school	Iquitos	M	Loreto	Maynas	San Juan	3°48′20′′S 73°19′26′′W	0	0	0	0	0	0	0	0	0
40	59,0	secondary school	Porvenir Pelejo San Martin	F	Loreto	Maynas	San Juan	3°48′20′′S 73°19′29′′W	0	0	0	0	0	0	0	0	0
41	15,0	Primary school	Iquitos	M	Loreto	Maynas	San Juan	3°48’36’’ S, 73°18’58’’ w	0	0	0	0	0	0	0	**Y**	15/12/2015
42	34,0	secondary school	Genaro Herrero	F	Loreto	Maynas	San Juan	3°48′28′′S 73°19′10′′W	0	0	0	0	0	0	0	0	0

Basic information (patient)					Address				Presence of triatomines						Chagas disease test		
House code	Age	Education level	Place of birth	Sex	Department	Province	District	Coordinates	Houses w/ triatomines intradom.	Triatomine intradom. Positive(N)	triatomines intradomocile negative(N)	Houses with triatomines peridomicile	triatomines peridomicile positive(N)	triatomines peridomicile negative(N)	Positive serology (blood donors)	Positive INS	Second test date (INS)
43	55,0	secondary school	Iquitos	M	Loreto	Maynas	San Juan	3°48′28′′S 73°19′10′′W	0	0	0	0	0	0	0	0	0
44	44,0	secondary school	Iquitos	F	Loreto	Maynas	San Juan	3°47′55′′S 73°18′57′′W	0	0	0	0	0	0	0	0	0
45	40,0	Primary school	Iquitos	F	Loreto	Maynas	San Juan	3°47′55′′S 73°18′57′′W	0	0	0	0	0	0	0	0	0
46	38,0	technical	Shapaja/ San Martin	F	Loreto	Maynas	Iquitos	3°75’64’’S 73°25’91’’W	0	0	0	0	0	0	0	**Y**	27/01/2015
47	10,0	Primary school	Pto Abeja	M	Loreto	Maynas	Mazan	3°10′31.77” S 73°12′53.86¨w	1	2	0	0	0	0	0	**Y**	23/01/2014
48	not found	-	Pto Abeja	-	-	-	-	-	-	-	-	-	-	-	Y	0	0
49	not found	-	-	-	-	-	Fernando Lores	-	-	-	-	-	-	-	0	**Y**	09/04/2012
50	63,0	Primary school	Padrecocha	F	Loreto	Maynas	Punchana	03°41.865′ S 073°16.720′ W	0	0	0	0	0	0	Y	0	0
51	61,0	Primary school	Padrecocha	F	Loreto	Maynas	Punchana	0341.885 07316795	0	0	0	0	0	0	0	0	0
52	39,0	secondary school	Nauta	M	Loreto	Maynas	Punchana	0341.906 ‘07316.758	0	0	0	0	0	0	0	0	0
53	59,0	secondary school	Padrecocha	F	Loreto	Maynas	Punchana	0341.933 07316.707	0	0	0	0	0	0	0	0	0
54	30,0	Primary school	Padrecocha	F	Loreto	Maynas	Punchana	0341.962 07316817	0	0	0	0	0	0	0	0	0
55	53,0	secondary school	Padrecocha	F	Loreto	Maynas	Punchana	03°41.963 073°16.774	0	0	0	0	0	0	0	0	0
56	41,0	secondary school	Iquitos	M	Loreto	Maynas	Punchana	034195 07316776	0	0	0	0	0	0	0	0	0
57	47,0	secondary school	Iquitos	M	Loreto	Maynas	Punchana	0342022 07316729	0	0	0	0	0	0	0	0	0
58	48,0	secondary school	Padrecocha	M	Loreto	Maynas	Punchana	0342.011 07316.736	0	0	0	0	0	0	0	0	0
59	31,0	secondary school	Fco. Orellana	F	Loreto	Maynas	Punchana	0	0	0	0	0	0	0	Y	N	19/08/2018
60	30,0	secondary school	Iquitos	M	Loreto	Maynas	Iquitos	0	0	0	0	0	0	0	0	0	0
61	65,0	secondary school	Yurimaguas	F	Loreto	Maynas	Punchana	0	0	0	0	0	0	0	0	0	0
62	-	-	Iquitos	-	-	-	-	not found	-	-	-	-	-	-	Y	0	0
63	24,0	University	Iquitos	F	Loreto	Maynas	Punchana	03.72 330°S 073.25 420W	0	0	0	0	0	0	0	N	20/09/2018
64	28,0	secondary school	Iquitos	F	Loreto	Maynas	Punchana	03.72 323°S 073.25 432W	0	0	0	0	0	0	0	0	0
65	40,0	secondary school	Iquitos	M	Loreto	Maynas	Punchana	03.72 333°S 073.25 441W	0	0	0	0	0	0	0	0	0
66	60,0	University	Lima	F	Loreto	Maynas	Iquitos	03.74312 073.25404	0	0	0	0	0	0	Y	0	0
67	41,0	secondary school	Mazan	F	Loreto	Maynas	Punchana	03.72320 073.26308	0	0	0	0	0	0	Y	0	0
68	-	-	Caserio Bellavista	-	-	-	-	not found	0	0	0	0	0	0	Y	0	0
69	-	-	Iquitos	-	-	-	-	3°75’341’’ S, 73°27’158’’ O	0	0	0	0	0	0	Y	0	0
70	51,0	secondary school	Yurimaguas	M	Loreto	Maynas	San Juan	3°74’414’’ S, 73°24’603’’ O	0	0	0	0	0	0	Y	0	0
71	55,0	secondary school	Iquitos	F	Loreto	Maynas	Iquitos	3.75564 07326154	0	0	0	0	0	0	Y	0	0
72	30,0	secondary school	Tamishiyacu	F	Loreto	Maynas	Iquitos	03.75623 075.25173	0	0	0	0	0	0	0	0	0
73	79,0	secondary school	Iquitos	M	Loreto	Maynas	Iquitos	03.75767 . 7326002	0	0	0	0	0	0	0	0	0
74	37,0	secondary school	Lagunas	M	Loreto	Maynas	Punchana	0372312 07326290	0	0	0	0	0	0	0	0	0
75	33,0	University	Iquitos	M	Loreto	Maynas	Iquitos	03.78393 073.28862	0	0	0	0	0	0	0	0	0
76	59,0	secondary school	Sauce	M	Loreto	Maynas	San Juan	3°46′1.32′S 73°16′37.60′′O	0	0	0	0	0	0	0	0	0
77	58,0	Primary school	Iquitos	F	Loreto	Maynas	San Juan	3°45′59.34′′S 73°16′37.42′′O	0	0	0	0	0	0	0	0	0
78	52,0	secondary school	Iquitos	F	Loreto	Maynas	San Juan	3°45′58.83′′ S 73°16′37.49′′O	0	0	0	0	0	0	0	0	0
79	68,0	Primary school	Apayacu- Yanashi	M	Loreto	Maynas	San Juan	3°54′6′′S 73°20′32′′W	0	0	0	0	0	0	0	0	0
80	60,0	Primary school	Pevas	M	Loreto	Maynas	San Juan	3°54′8′′S 73°20′30′′W	0	0	0	0	0	0	0	0	0
81	36,0	Primary school	Iquitos	F	Loreto	Maynas	San Juan	3°54′15′′S 73°20′29′′W	0	0	0	0	0	0	0	0	0
82	75,0	Primary school	Iquitos	F	Loreto	Maynas	San Juan	3°54′16′′S 73°20′28′′W	0	0	0	0	0	0	0	0	0
83	43,0	Primary school	Iquitos	M	Loreto	Maynas	San Juan	0	0	0	0	0	0	0	0	0	0
84	39,0	Primary school	Iquitos	M	Loreto	Maynas	San Juan	0	0	0	0	0	0	0	0	0	0
85	35,0	secondary school	Iquitos	F	Loreto	Maynas	San Juan	0	0	0	0	0	0	0	0	0	0
86	48,0	Primary school	Iquitos	F	Loreto	Maynas	San Juan	0	0	0	0	0	0	0	0	0	0

Basic information (patient)					Address				Presence of triatomines						Chagas disease test		
House code	Age	Education level	Place of birth	Sex	Department	Province	District	Coordinates	Houses w/ triatomines intradom.	Triatomine intradom. Positive(N)	triatomines intradomocile negative(N)	Houses with triatomines peridomicile	triatomines peridomicile positive(N)	triatomines peridomicile negative(N)	Positive serology (blood donors)	Positive INS	Second test date (INS)
87	50,0	Primary school	Iquitos	M	Loreto	Maynas	San Juan	0	0	0	0	0	0	0	0	0	0
88	not found	-	Iquitos	-	-	-	-	-	-	-	-	-	-	-	Y	0	0
89	not found	-	Iquitos	-	-	-	-	3°73'921'' S, 73°24'725'' O	-	-	-	-	-	-	Y	0	0
90	not found	-	Nueva Anguilla	-	-	-	-	0	-	-	-	-	-	-	Y	0	0
91	not found	-	Tamichiyacu	-	-	-	-	0	-	-	-	-	-	-	Y	0	0
92	not found	-	Caserio Sta Rosa-Rio Napo	-	-	-	-	3°74'832'' S, 73°26'424'' O	-	-	-	-	-	-	Y	0	0
93	not found	-	Iquitos	-	-	-	-	3°75'623'' S, 73°24'905'' O	-	-	-	-	-	-	Y	0	0
94	not found	-	Santa Maria	-	-	-	-	0	-	-	-	-	-	-	Y	0	0
95	not found	-	Yaquerana	-	-	-	-	0	-	-	-	-	-	-	Y	0	0
96	not found	-	0	-	-	-	-	0	-	-	-	-	-	-	0	0	0
97	63	Primary school	San Martin	F	Loreto	Maynas	Iquitos	0	1	0	3	0	0	0	0	0	0
98	44	secondary school	Caserio Loboyacu	M	Loreto	Maynas	Iquitos	0	1	2	0	1	1	0	0	0	0
99	48	Primary school	Caserio Loboyacu	F	Loreto	Maynas	Iquitos	0	0	0	0	0	0	0	0	0	0
100	43	secondary school	Caserio Loboyacu	F	Loreto	Maynas	Iquitos	0	1	1	0	0	0	0	0	0	0
101	20	secondary (unfinished)	Yurimaguas	F	Loreto	Maynas	San Juan	03.78.522, 73.33.105	0	0	0	0	0	0	0	0	0
102	29	Primary school	Nauta	F	Loreto	Maynas	Punchana	3°43'6.28´´S 73°14'32.84´´O	0	0	0	0	0	0	0	0	0
103	39	secondary school	Iquitos	F	Loreto	Maynas	Iquitos	0	0	0	0	1	9	2	0	0	0
104	39	Primary school	Caserio Loboyacu	F	Loreto	Maynas	Iquitos	0	1	3	0	0	0	0	0	0	0
105	51	Primary school	Centro Yacapana-Tamishiyacu	F	Loreto	Maynas	Iquitos	S 03.74966 , W 073.39057	1	1	0	0	0	0	0	0	0
106	37	Primary school	Caserio Loboyacu	F	Loreto	Maynas	Iquitos	S 03.74797, W 073.39123	1	4	0	0	0	0	0	0	0
107	34	secondary school	Genaro Herrero	M	Loreto	Maynas	San Juan	03.78.478, 73.33.195	0	0	0	0	0	0	0	0	0
108	51,0	Primary school	Yarina	F	Loreto	Maynas	San Juan	0	0	0	0	0	0	0	0	0	0
109	58,0	secondary school	Sta Clara	M	Loreto	Maynas	San Juan	3.78.16, 73.33.177	0	0	0	0	0	0	0	0	0
110	60,0	University	Iquitos	M	Loreto	Maynas	San Juan	3.78.454, 73.33.198	0	0	0	0	0	0	0	0	0
111	36,0	Primary school	Datem del Marañon	M	Loreto	Maynas	San Juan	0	0	0	0	0	0	0	0	0	0
112	56,0	Primary school	Iquitos	M	Loreto	Maynas	San Juan	03.78.269, 73.32.340	0	0	0	0	0	0	0	0	0
113	25,0	secondary school	Iquitos	F	Loreto	Maynas	San Juan	03.78.486, 73.33.124	0	0	0	0	0	0	0	0	0
114	79,0	secondary school	Iquitos	M	Loreto	Maynas	San Juan	03.78.284, 73.32.069	0	0	0	0	0	0	0	0	0
115	48,0	secondary school	Iquitos	F	Loreto	Maynas	San Juan	03.78.605 , 73.33.144	0	0	0	0	0	0	0	0	0
116	70,0	University	Iquitos	M	Loreto	Maynas	San Juan	03.78.609, 73.33.173	0	0	0	0	0	0	0	0	0
117	33,0	University	Iquitos	M	Loreto	Maynas	San Juan	03.78.269, 73.32.345	0	0	0	0	0	0	0	0	0
118	50,0	Primary school	Iquitos	F	Loreto	Maynas	San Juan	03.80.687, 73.33.945	0	0	0	0	0	0	0	0	0
119	47,0	secondary school	Iquitos	F	Loreto	Maynas	San Juan	03.80.605, 73.33.889	0	0	0	0	0	0	0	0	0
120	67,0	Primary school	Iquitos	F	Loreto	Maynas	San Juan	03.80.614, 73.33.906	0	0	0	0	0	0	0	0	0
121	43,0	Primary school	Iquitos	M	Loreto	Maynas	San Juan	03.48.408, 73.20.502	0	0	0	0	0	0	0	0	0
122	36,0	secondary (unfinished)	Iquitos	F	Loreto	Maynas	San Juan	03.48.409, 73.20.502	0	0	0	0	0	0	0	0	0
123	47,0	Primary school	Iquitos	F	Loreto	Maynas	San Juan	03.48.382, 73.20.443	0	0	0	0	0	0	0	0	0
124	54,0	Primaria	Iquitos	F	Loreto	Maynas	San Juan	03.78.395, 73.33.678	0	0	0	0	0	0	0	0	0
125	70,0	secondary school	Iquitos	M	Loreto	Maynas	San Juan	03.80.676, 73.34.180	0	0	0	0	0	0	0	0	0
126	37,0	secondary school	Tamishiyacu	F	Loreto	Maynas	San Juan	03.80.722, 73.33.862	0	0	0	0	0	0	0	0	0
127	36,0	secondary school	Juanjui-San Martin	F	Loreto	Maynas	San Juan	03.80.781, 73.33.791	0	0	0	0	0	0	0	0	0
128	66,0	Primary school	Iquitos	M	Loreto	Maynas	San Juan	03.80.997, 73.33.659	0	0	0	0	0	0	0	0	0
129	32,0	Primary school	Comunidad Huanta- Bajo Amazonas	Iquitos	Loreto	Maynas	San Juan	03.80.214, 73.33.869	0	0	0	0	0	0	0	0	0
130	29,0	Primary school	Iquitos	Iquitos	Loreto	Maynas	San Juan	3.78363, 73.33907	0	0	0	0	0	0	0	0	0
131	23	University (unfinished)	Iquitos	F	Loreto	Maynas	San Juan	03°80'70" S, 73°34'13''W	0	0	0	1	1	0	0	0	0

Basic information (patient)					Address				Presence of triatomines						Chagas disease test		
House code	Age	Education level	Place of birth	Sex	Department	Province	District	Coordinates	Houses w/ triatomines intradom.	Triatomine intradom. Positive(N)	triatomines intradomocile negative(N)	Houses with triatomines peridomicile	triatomines peridomicile positive(N)	triatomines peridomicile negative(N)	Positive serology (blood donors)	Positive INS	Second test date (INS)
132	20	secondary school	San Juan	M	Loreto	Maynas	San Juan	0380728 07334132	0	0	0	0	0	0	0	0	0
133	48,0	secondary school	San Juan	M	Loreto	Maynas	San Juan	0380172 07333854	0	0	0	0	0	0	0	0	0
134	50,0	Primary school	Rio Ucayali	F	Loreto	Maynas	San Juan	0380224 07333887	0	0	0	0	0	0	0	0	0
135	46,0	Primary school	Caserio Buenos Aires-Nauta	M	Loreto	Maynas	Iquitos	03 45 980 073 22 548	0	0	0	0	0	0	0	0	0
136	27,0	Primary school	Iquitos	F	Loreto	Maynas	Iquitos	03° 46 101 073 22 410	0	0	0	0	0	0	0	0	0
137	24,0	secondary school	Iquitos	F	Loreto	Maynas	Iquitos	0	0	0	0	0	0	0	0	0	0
138	27,0	secondary school	Iquitos	F	Loreto	Maynas	Iquitos	03° 45 971 073 22 547	0	0	0	0	0	0	0	0	0
139	50,0	Primary school	Iquitos	F	Loreto	Maynas	Iquitos	03° 46 100 073 22 398	0	0	0	0	0	0	0	0	0
140	50,0	Primary school	Iquitos	F	Loreto	Maynas	Iquitos	03° 46 125 073 22 358	0	0	0	0	0	0	0	0	0
141	38,0	secondary school	Iquitos	F	Loreto	Maynas	Iquitos	0345942 07322523	0	0	0	0	0	0	0	0	0
142	72,0	Primary school	Alto Amazonas	M	Loreto	Maynas	Iquitos	03°46′114′S 073°22.368′O	0	0	0	0	0	0	0	0	0

INS: Instituto de Salud del Perú; F: female; M: male; Y: confirmed Chagas disease cases.

TABLE II

Description of the households for Chagas disease vector monitoring in Iquitos, Peru, for statistical analysis

House	Roof	Wall material	Floor material	Cracks observed	Mattress material	Palms nearby	Infested houses	Domestic triatomines observed by owner	Poses animals	Animal nests	Surrounding scrublands	Outdoor animals present	Presence of trees	Rustic houses nearby
1	calamine	wood	wood	1	foam	0	0	0	0	0	0	0	1	1
3	palm	wood	earth	1	no mattress	1	0	1	0	0	1	0	1	1
4	calamine	brick	concrete	1	spring	0	0	1	1	0	0	0	1	0
6	calamine	brick	concrete	0	spring	0	0	0	0	0	0	0	0	0
7	palm	costal	concrete	1	thatch	0	0	0	1	0	1	1	1	0
8	calamine	brick	concrete	1	thatch	1	0	0	1	0	1	0	1	1
14	calamine	brick	concrete	0	spring	0	0	0	1	0	0	0	0	0
15	calamine	wood	concrete	1	no mattress	1	0	0	1	0	1	1	1	1
16	calamine	brick	concrete	0	spring	0	0	0	0	0	0	0	0	0
17	calamine	wood	wood	0	foam	0	0	0	1	0	1	0	0	1
20	calamine	wood	concrete	0	foam	0	0	0	1	0	1	1	0	1
22	calamine	brick	concrete	1	spring	0	0	0	0	0	0	0	0	0
25	calamine	wood	concrete	1	spring	1	0	0	1	0	1	0	1	1
26	calamine	brick	earth	1	spring	1	0	0	1	1	1	1	1	1
27	calamine	wood	earth	1	thatch	1	0	0	1	1	1	1	1	1
28	palm	wood	concrete	1	thatch	1	0	0	1	0	1	1	1	1
29	calamine	brick	concrete	0	spring	1	0	0	1	0	0	0	1	0
31	calamine	wood	wood	1	foam	0	0	0	1	1	1	1	0	1
34	calamine	brick	concrete	1	no mattress	1	0	0	1	1	1	1	1	1
35	palm	wood	wood	1	no mattress	1	0	0	1	1	1	1	1	1
36	calamine	brick	concrete	1	spring	0	0	0	1	0	0	0	1	1
37	calamine	brick	concrete	1	foam	1	0	0	1	1	0	0	1	1
38	calamine	wood	earth	1	foam	1	0	0	1	1	1	1	1	1
39	calamine	wood	wood	1	thatch	1	0	0	1	1	1	1	1	1
40	calamine	wood	earth	1	foam	1	0	0	1	1	1	1	1	1
41	calamine	brick	concrete	1	foam	1	0	0	1	0	1	1	1	1

House	Roof	Wall material	Floor material	Cracks observed	Mattress material	Palms nearby	Infested houses	Domestic triatomines observed by owner	Poses animals	Animal nests	Surrounding scrublands	Outdoor animals present	Presence of trees	Rustic houses nearby
42	calamine	wood	wood	1	foam	1	0	0	1	1	1	1	1	1
43	calamine	brick	concrete	1	spring	1	0	0	1	1	1	1	1	1
44	calamine	wood	wood	1	spring	1	0	0	1	1	1	1	1	1
45	calamine	wood	earth	1	thatch	1	0	0	0	1	1	1	1	1
46	calamine	wood	concrete	1	spring	1	0	0	0	0	0	0	1	0
47	palm	wood	wood	1	no mattress	1	1	1	1	1	1	1	1	1
50	calamine	brick	concrete	1	thatch	0	0	0	1	0	0	0	1	1
51	calamine	brick	concrete	1	thatch	1	0	0	0	1	1	1	1	1
52	calamine	brick	concrete	0	spring	1	0	0	0	1	1	1	1	1
53	calamine	brick	concrete	0	spring	1	0	0	0	1	1	1	1	1
54	calamine	wood	wood	1	spring	1	0	0	1	1	1	1	1	1
55	calamine	brick	concrete	1	spring	0	0	0	1	0	0	1	1	1
56	calamine	brick	concrete	1	spring	1	0	0	1	0	0	0	1	1
57	calamine	wood	earth	1	thatch	1	0	0	1	1	1	1	1	1
58	calamine	brick	concrete	1	thatch	0	0	0	1	0	1	1	1	1
59	calamine	wood	wood	1	spring	0	0	0	0	1	0	1	0	1
60	calamine	brick	concrete	0	spring	0	0	0	1	0	0	0	0	0
61	calamine	wood	wood	1	spring	0	0	0	1	1	1	1	0	1
63	calamine	wood	concrete	1	foam	0	0	0	1	0	0	0	0	1
64	calamine	wood	earth	1	foam	0	0	0	0	0	1	1	0	1
65	calamine	wood	earth	1	spring	0	0	0	0	0	1	1	0	1
66	calamine	brick	concrete	0	spring	1	0	0	0	1	1	1	1	0
67	calamine	brick	concrete	0	spring	0	0	1	0	1	0	1	0	1
70	calamine	brick	concrete	1	spring	1	0	0	1	1	1	1	1	1
71	calamine	brick	concrete	0	spring	0	0	0	0	0	0	0	1	1
72	calamine	wood	earth	1	foam	1	0	0	0	1	1	1	1	1
73	calamine	brick	concrete	0	spring	1	0	1	0	1	1	1	1	1
74	calamine	wood	earth	1	spring	0	0	0	1	0	0	0	0	1
75	calamine	brick	concrete	1	spring	1	0	0	0	1	1	1	1	1
76	calamine	brick	concrete	0	spring	1	0	0	1	1	1	1	1	0
77	calamine	wood	concrete	1	foam	0	0	1	0	0	0	0	1	0
78	calamine	wood	concrete	1	foam	1	0	0	1	0	1	1	1	0
79	palm	wood	wood	1	foam	1	0	1	0	1	1	1	1	1
80	calamine	wood	earth	1	no mattress	1	0	1	1	1	1	1	1	1
81	calamine	wood	wood	1	thatch	1	0	1	1	1	1	1	1	1
82	calamine	brick	concrete	1	foam	1	0	0	1	1	1	1	1	1
83	calamine	brick	concrete	1	foam	0	0	0	1	0	0	1	1	1
84	palm	wood	earth	1	thatch	1	0	0	1	1	1	1	1	1
85	calamine	wood	earth	1	thatch	0	0	0	1	0	1	1	1	1
86	calamine	brick	concrete	1	foam	1	0	0	1	1	1	1	1	1
87	palm	wood	earth	1	thatch	1	0	0	1	1	0	1	1	1
97	palm	wood	wood	1	no mattress	1	1	1	1	1	1	1	1	1
98	palm	wood	wood	1	no mattress	1	1	1	1	1	1	1	1	1
99	calamine	wood	wood	1	no mattress	1	0	1	0	1	1	1	1	1
100	palm	wood	wood	1	no mattress	1	1	1	0	1	1	1	1	1
101	calamine	wood	earth	1	foam	1	0	0	0	0	1	1	1	1
102	calamine	brick	concrete	1	thatch	0	0	0	0	0	0	0	0	1
103	calamine	wood	wood	1	no mattress	1	0	0	1	1	1	1	1	1

House	Roof	Wall material	Floor material	Cracks observed	Mattress material	Palms nearby	Infested houses	Domestic triatomines observed by owner	Poses animals	Animal nests	Surrounding scrublands	Outdoor animals present	Presence of trees	Rustic houses nearby
104	calamine	wood	wood	1	thatch	1	1	1	0	1	1	1	1	1
105	palm	wood	wood	1	no mattress	1	1	1	0	1	1	1	1	1
106	palm	wood	wood	1	no mattress	1	1	1	1	1	1	1	1	1
107	calamine	wood	earth	1	thatch	1	0	1	1	1	1	0	1	0
108	calamine	wood	wood	1	no mattress	1	0	1	0	0	1	1	1	1
109	calamine	without walls	earth	1	no mattress	1	0	0	0	1	1	1	1	1
110	calamine	brick	concrete	1	spring	1	0	0	1	1	1	1	1	1
111	calamine	wood	wood	1	no mattress	1	0	0	1	1	1	1	1	0
112	calamine	wood	earth	1	no mattress	1	0	0	1	1	1	1	1	1
113	calamine	wood	earth	1	thatch	0	0	0	1	0	1	1	1	1
114	palm	wood	earth	1	thatch	1	0	0	1	0	0	0	0	0
115	calamine	brick	concrete	1	spring	1	0	0	1	1	1	1	1	1
116	calamine	brick	concrete	1	foam	1	0	0	1	1	1	1	1	1
117	calamine	wood	wood	1	thatch	1	0	1	1	1	1	1	1	1
118	calamine	wood	earth	1	thatch	1	0	0	1	1	1	1	1	1
119	calamine	wood	earth	1	foam	1	0	0	1	1	1	1	1	1
120	calamine	wood	earth	1	foam	1	0	0	1	1	1	1	1	1
121	calamine	wood	concrete	1	no mattress	1	0	0	0	1	1	0	1	1
122	calamine	wood	concrete	1	foam	1	0	1	1	1	1	1	1	1
123	calamine	wood	concrete	1	foam	1	0	1	0	0	1	1	1	1
124	calamine	brick	concrete	1	spring	1	0	0	1	1	1	1	1	1
125	calamine	wood	concrete	1	foam	1	0	0	1	1	1	1	1	1
126	palm	wood	earth	1	spring	1	0	0	1	1	1	1	1	1
127	calamine	wood	earth	1	foam	1	0	0	1	1	1	1	1	1
128	palm	wood	concrete	1	spring	1	0	1	1	1	1	1	1	1
129	calamine	wood	wood	1	thatch	1	0	1	1	1	1	1	1	1
130	calamine	wood	earth	1	foam	1	0	1	1	1	1	1	1	1
131	calamine	wood	earth	1	spring	1	0	1	1	1	1	1	1	1
132	calamine	brick	concrete	1	spring	1	0	1	1	1	1	1	1	1
133	calamine	brick	concrete	1	spring	1	0	0	1	0	1	1	1	1
134	calamine	wood	concrete	1	spring	1	0	0	1	1	1	1	1	1
135	calamine	wood	earth	1	foam	1	0	1	1	1	1	1	1	1
136	calamine	wood	wood	1	foam	1	0	1	1	1	1	1	1	1
137	palm	wood	wood	1	foam	1	0	1	1	1	1	1	1	1
138	calamine	wood	wood	1	foam	1	0	1	1	1	1	1	1	1
139	calamine	wood	earth	1	foam	1	0	0	1	1	1	1	1	1
140	calamine	wood	concrete	1	foam	1	0	0	1	1	1	1	1	1
141	calamine	wood	wood	1	no mattress	1	0	0	0	1	1	1	1	1
142	palm	wood	earth	1	thatch	1	0	1	0	1	1	1	1	1
